# Increased Angiopoietin-1 and -2 levels in human vitreous are associated with proliferative diabetic retinopathy

**DOI:** 10.1371/journal.pone.0280488

**Published:** 2023-01-20

**Authors:** Teresa Tsai, Mohannad Alwees, Mohammad Ali Asaad, Janine Theile, Vinodh Kakkassery, H. Burkhard Dick, Tim Schultz, Stephanie C. Joachim

**Affiliations:** 1 Experimental Eye Research Institute, University Eye Hospital, Ruhr-University Bochum, Bochum, Germany; 2 Department of Ophthalmology, University of Luebeck, Luebeck, Germany; Boston University School of Medicine, UNITED STATES

## Abstract

**Background:**

Diabetic retinopathy is a frequent complication of diabetes mellitus and a leading cause of blindness in adults. The objective of this study was to elucidate the diabetic retinopathy pathophysiology in more detail by comparing protein alterations in human vitreous of different diabetic retinopathy stages.

**Methods:**

Vitreous samples were obtained from 116 patients undergoing pars plana vitrectomy. Quantitative immunoassays were performed of angiogenic factors (VEGF-A, PIGF, Angiopoietin-1, Angiopoietin-2, Galectin-1) as well as cytokines (IL-1β, IL-8, IFN-γ, TNF-α, CCL3) in samples from control patients (patients who don’t suffer from diabetes; n = 58) as well as diabetes mellitus patients without retinopathy (n = 25), non-proliferative diabetic retinopathy (n = 12), and proliferative diabetic retinopathy patients (n = 21). In addition, correlation analysis of protein levels in vitreous samples and fasting glucose values of these patients as well as correlation analyses of protein levels and VEGF-A were performed.

**Results:**

We detected up-regulated levels of VEGF-A (p = 0.001), PIGF (p<0.001), Angiopoietin-1 (p = 0.005), Angiopoietin-2 (p<0.001), IL-1β (p = 0.012), and IL-8 (p = 0.018) in proliferative diabetic retinopathy samples. Interestingly, we found a strong positive correlation between Angiopoietin-2 and VEGF-A levels as well as a positive correlation between Angiopoietin-1 and VEGF-A.

**Conclusion:**

This indicated that further angiogenic factors, besides VEGF, but also pro-inflammatory cytokines are involved in disease progression and development of proliferative diabetic retinopathy. In contrast, factors other than angiogenic factors seem to play a crucial role in non-proliferative diabetic retinopathy development. A detailed breakdown of the pathophysiology contributes to future detection and treatment of the disease.

## Introduction

Diabetic retinopathy (DR) occurs as a secondary disease of diabetes mellitus (DM), affects adults aged 20–74 in developed countries, and is one of the most common causes for new cases of blindness [1]. As a result, patients who are already impacted by DM are confronted with the social as well as the economic burden of visual impairment and blindness through DR [2].

DR can be subdivided into different stages, starting with the early stage of mild non-proliferative DR (NPDR) and further progressing to the proliferative DR (PDR) stage. NPDR is mainly characterized by vascular changes and circulatory defects, whereas PDR is characterized by pathological preretinal neovascularization [3, 4]. Based on the various DR stages, which are associated with different mechanisms or factors that contribute to its development, it is crucial to take these stages into account when studying DR.

As in other neurodegenerative diseases, inflammation plays a pivotal role in DR pathogenesis. By upregulating various inflammatory factors, phagocytic monocytes/macrophages and microglial cells are recruited, which in turn release various cytotoxic cytokines [5, 6]. Cytokines that mainly seem to play a role in the development of retinopathy in DM are the pro-inflammatory cytokines interleukin-1β (IL-1β), IL-6, and tumor necrosis factor (TNF)-α, as well as the chemokine IL-8 as these are elevated in vitreous samples from PDR patients [7–11]. In our previous study, we were also able to detect an up-regulation of the pro-inflammatory cytokines IL-1ß and IFN-γ but the pro-inflammatory cytokine IL-6 as well as the anti-inflammatory cytokines IL-2, IL-4, and IL-13 were not altered in the vitreous of DR patients [12]. In this previous study we did not differentiate between different DR stages.

In addition to inflammatory processes neovascularization is the main hallmark of DR pathogenesis and VEGF is postulated to be a key regulator of PDR [13]. In addition to VEGF, recent research has shown that the angiopoietin family also plays an important role in regulating the growth of new blood vessels [14]. In this process Angiopoietin-1 and 2 are acting agonistically and antagonistically [15]. Angiopoietin-1, predominantly promotes endothelial cell survival *in vitro* [16] and dose-dependently blocks diabetic damage to the blood-retinal barrier [17], suggesting that Angiopoietin-1 has a protective effect against DR. In contrast, Angiopoietin-2 is up-regulated under pathological conditions and seems to be a cooperative driver of angiogenesis and vascular destabilization along with VEGF [18–20]. Therefore, it is not surprising that Angiopoietin-2 is elevated in serum of PDR patients [21] and seems to support DR progression.

In addition to Angiopoietins, Galectin-1 also plays an important and regulatory role in the proper execution of the angiogenesis process, such that loss of endogenous Galectin-1 in endothelial cells leads to impaired angiogenesis [22–24]. Recent studies further suggest that Galectin-1 is also involved in the pathogenesis of PDR. Thus, a significant increase of Galectin-1 as well as a positive correlation between Galectin-1 and VEGF levels were found in vitreous samples from PDR patients compared to non-diabetic patients [22, 25].

Several potential DR therapies have been investigated in recent years. By now, anti-VEGF therapy has become the first-line therapy. However, many treatments are often required over months and years to achieve successful treatment. Moreover, in about 30% of DR patients it can be observed that they do not respond to initial anti-VEGF treatment [13], which underpins the importance of other approaches such as targeting inflammatory cytokines.

This raised the question of what other factors are involved in the development of DR and to what extent protein changes occur during DR progression. Hence, the aim of our study was to analyze protein alterations in vitreous samples of patients with different stages of DR, more precisely with NPDR and PDR, in comparison to DM patients without DR and controls (patients with macular hole or macular pucker undergoing vitrectomy) to reach a better understanding of DR pathophysiology. The gained understanding can lead to the development of further additive treatment options and a more precise adjustment of therapies to the DR course or form, which would support an individual treatment of the disease and significantly improve the overall quality of life.

## Materials and methods

### Subjects, clinical examinations, and sample collection

All patients gave their written informed consent before the start of the study as previously described and the Declaration of Helsinki was observed [12, 26]. Approval for this study was granted by the local Ethics Committee of the Ruhr-University Bochum (Bochum, Germany; approval number: 15–5363).

Prior to sample collection, patients were divided in four groups based on the clinical examination. The classification was based on the modified Arlie-House classification, also used in the Early Treatment in Diabetic Retinopathy Studies (ETDRS): Controls, diabetes mellitus (DM), non-proliferative diabetic retinopathy (NPDR), and proliferative diabetic retinopathy (PDR). For the first three groups, there was an indication for vitrectomy other than diabetic retinopathy, for example macular hole or macular pucker. For every patient clinical data, including mean age, gender, eye, DR stadium, fasting glucose, as well as HbA1c, were collected (Table 2). Patient with an age under 21, with a presence of glaucoma, or with previous vitrectomy were excluded from this study.

The study originally included 135 patients (27 patients per group). However, vitreous samples that were too small for the planned ELISA analysis were excluded. Therefore, vitreous samples (1 ml/patient) from 25 patients with DM, 12 patients with NPDR, and 21 patients with PDR were collected by transconjunctival pars plana vitrectomy using a 23-gauge cutter. For this purpose, a core vitrectomy was performed with the gauge cutter after insertion of the trocars and before turning on the infusion. A 10 ml microsyringe connected to the aspiration tube of the cutter was used to collect the vitreous sample. The samples were immediately frozen and stored at -80°C until analysis.

### Measurement of cytokines in vitreous samples

All proteins in vitreous samples were quantified using commercially available enzyme-linked immunosorbent assay kits (ELISA; Table 1). Each assay was performed according to the manufacturer’s instructions as previously described [26]. Samples for measurements of CCL3 and galectin-1 were diluted with sample dilution buffer immediately before the assay as analyzed (Table 1). Vitreous samples for all other measurements were used without dilution. The subsequent analyses were performed on a microplate reader (AESKU Reader with Gen5 ELISA Software, AESKU. DIAGNOSTICS, Wendelsheim, Germany) [12].

**Table 1:** Applied ELISA assays including company, catalogue number, dilution, and references.

Protein	Company	Catalogue number	Dilution	Reference
Angiopoietin-1	R&D Systems	DANG10	undiluted	[27]
Angiopoietin-2	R&D Systems	DANG20	undiluted	[27]
CCL3/MIP-1α	R&D Systems	DMA00	1:50	[28]
Galectin-1	R&D Systems	DGAL10	1:20	[29]
IFN-γ	Invitrogen	BMS228	undiluted	[12]
IL-1β/IL-1F2	R&D Systems	DLB50	undiluted	[12]
IL-8/CXCL8	R&D Systems	D8000C	undiluted	[30]
TNF-α	R&D Systems	DTA00D	undiluted	[31]
PIGF	R&D Systems	DPG00	undiluted	[12]
VEGF-A	Invitrogen	BMS277-2	undiluted	[12, 32]

### Statistical analysis

As in previous studies, a commercial predictive analysis program (version 13.3; Statistica, Tulsa, OK, USA) was used to perform the statistical analyses [26]. ANOVA followed by Tukey post-hoc test was applied to determine significant differences in vitreous protein concentrations among the four groups. P-values below 0.05 were considered to be statistically significant with *p<0.05, **p<0.01, and ***p<0.001. All graphs display mean values±standard error (SEM)±standard deviation (SD).

The Pearson’s correlation coefficient (r) was calculated between patient’s individual protein levels and patient’s fasting glucose level or VEGF-A level.

## Results

### Preoperative clinical data of the patients

In total, 116 vitreous samples were analyzed, 61 from male and 55 from female patients. The patients in the control group had a mean age of 77.05±9.01 years and the DM patients a mean age of 80.11±6.98 years. The DR group was subdivided into NDPR and PDR patients. The NDPR patients had a mean age of 71.66±8.99 years and the PDR group of 62.26±14.42 years. Therefore, the control and DM patients were significantly older than the PDR patients (PDR vs. control or vs. DM: p<0.001).

Regarding gender and side ratio of the operated eye no significant differences were detectable between all four groups (p>0.05).

Moreover, laboratory values, which provide diabetic indications, were collected. The patients in the control group had a mean fasting glucose level of 77.05±9.01 mg/dl and a mean HbA1c value of 5.65±0.62. Both indicators were significantly higher in the DM, the NPDR, and the PDR group (all: p<0.001; Table 2).

**Table 2:** Clinical patient data for all groups. Abbreviations: y = year; SD = standard deviation; M = male; F = female; OD = right eye; OS = left eye.

	Controls	DM	NPDR	PDR
**Samples per group**	58	25	12	21
**Mean age ± SD (y)**	77.05±9.01	80.11±6.98	71.66±8.99	62.26±14.42
**Gender (M/F)**	29/29	14/11	5/7	13/8
**Eye (OD/OS)**	26/32	13/12	7/5	10/11
**Fasting glucose (mg/dl)**	111.63±31.97	158.35±59.97	174.62±45.24	180.81±61.85
**HbA1c ± SD**	5.65±0.62	7.19±1.40	7.13±0.93	8.10±1.46

### Strong up-regulation of angiogenic factors in PDR patients

**Fig 1:**
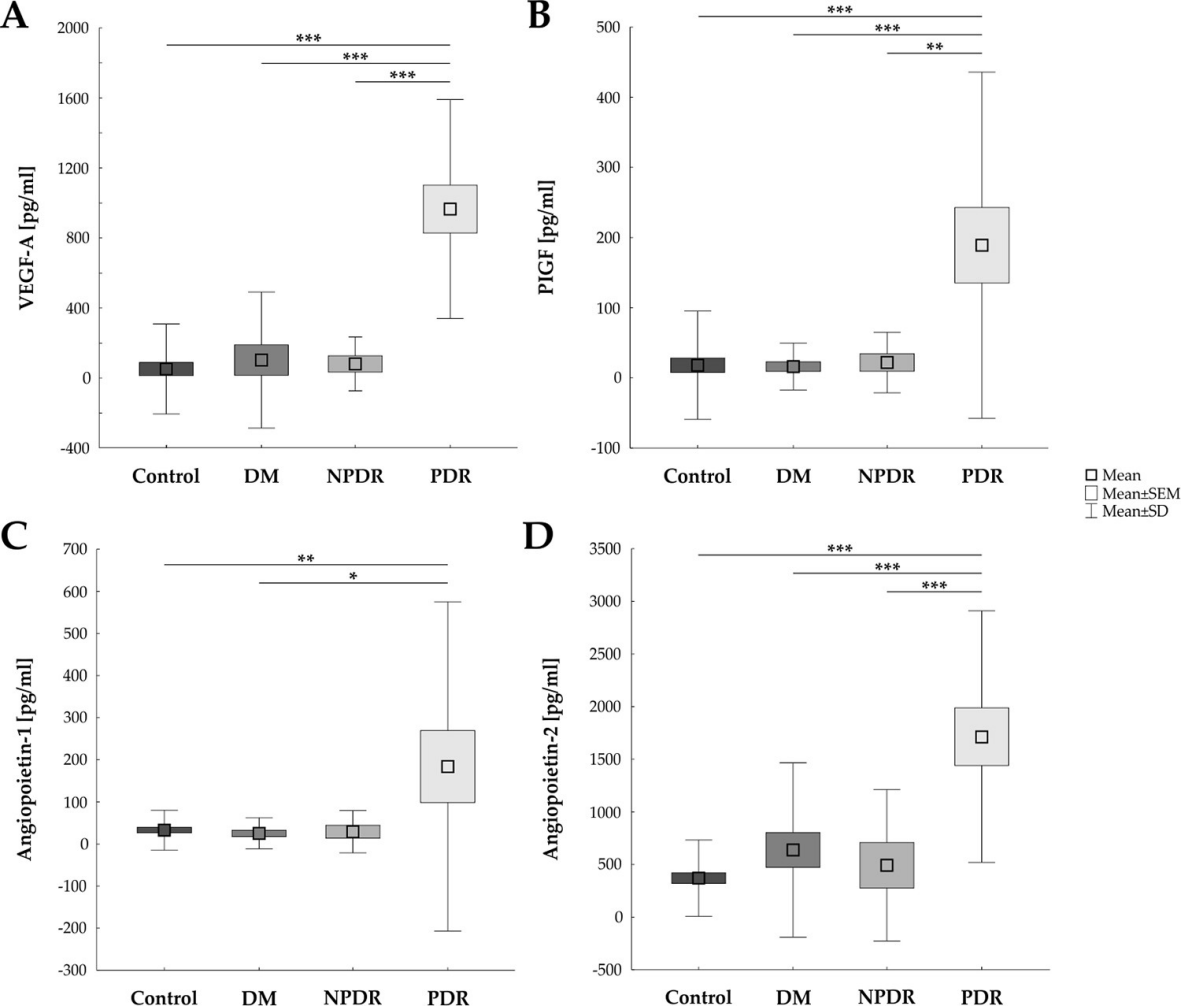
Up-regulation of different angiogenic factors in vitreous samples. **A)** VEGF-A was significantly up-regulated in vitreous samples of PDR patients in comparison to all other analyzed groups (all: p<0.001). **B)** Additionally, PIGF was elevated in the PDR group in contrast to the control (p<0.001), the DM (p<0.001), and the NPDR group (p = 0.001). **C)** Angiopoietin-1 was increased in PDR samples compared to control (p = 0.005) and DM samples (p = 0.013). **D)** Angiopoietin-2 was also elevated in the PDR group in contrast to the other study groups (all: p<0.001). DM: diabetes mellitus; NPDR: non-proliferative diabetic retinopathy; PDR: proliferative diabetic retinopathy (PDR); values are mean ± SEM ± SD; *p<0.05; **p<0.01; ***p<0.001.

Compared to controls, vitreous samples of PDR patients revealed an up-regulation of all analyzed angiogenic factors (Fig 1 and Table 3). The mean level of VEGF-A in vitreous samples of PDR patients (970.55±625.33 pg/ml) was significantly higher than in control patients (57.07±257.30 pg/ml; p<0.001). Also, in comparison to the DM group (108.23±388.35 pg/ml; p<0.001) and NPDR group (86.18±153.96 pg/ml; p<0.001), the level of VEGF-A was up-regulated (Fig 1A).

**Table 3:** Cytokine concentration (mean ± SEM) in vitreous samples of control, diabetes mellitus (DM), non-proliferative diabetic retinopathy (NPDR) patients, and proliferative diabetic retinopathy (PDR) patients measured via ELISA. Significant p-values are in bold.

	Cytokine concentration				P-value					
Cytokine	Control	DM	NPDR	PDR	DM *vs*. control	NPDR *vs*. control	PDR *vs*. control	NPDR *vs*. DM	PDR *vs*. DM	PDR *vs*. NPDR
**Angiopoietin-1 [pg/ml]**	32.48±47.51	25.39±36.92	29.19±50.36	184.01±391.08	1.000	1.000	**0.005**	1.000	**0.013**	0.083
**Angiopoietin-2 [pg/ml]**	370.70±362.42	638.47±828.36	492.46±719.90	1714.85±1194.35	0.424	0.957	**<0.001**	0.944	**<0.001**	**<0.001**
**CCL3/MIP-α [pg/ml]**	178.82±235.57	65.22±163.81	71.74±162.59	89.55±141.27	0.122	0.373	0.317	1.000	0.978	0.995
**Galectin-1 [ng/ml]**	6.10±6.64	5.99±6.47	7.73±7.36	10.77±4.89	1.000	0.891	0.053	0.897	0.099	0.600
**IL-1β [pg/ml]**	0.22±0.56	0.23±0.42	0.17±0.22	0.78±1.23	1.000	1.000	**0.012**	0.994	**0.043**	0.090
**IL-8 [pg/ml]**	39.99±53.25	82.59±107.41	20.67±9.43	101.49±79.04	0.204	0.892	**0.018**	0.170	0.858	**0.034**
**PIGF [pg/ml]**	18.10 ±77.28	16.20±33.60	22.00±43.19	189.12±246.55	1.000	1.000	**<0.001**	1.000	**<0.001**	**0.001**
**VEGF-A [pg/ml]**	57.07±257.30	108.23±388.35	86.18±153.96	970.55±625.33	0.9596	0.989	**<0.001**	0.999	**<0.001**	**<0.001**

Furthermore, the level of PIGF was significantly up-regulated in PDR patients (189.12±246.55 pg/ml) in comparison to the controls (18.10±77.28 pg/ml; p<0.001) as well as to DM (16.20±33.60 pg/ml; p<0.001) and to NPDR samples (22.00±43.19 pg/ml; p = 0.001; Fig 1B).

Additionally, the Angiopoietin-1 level was elevated in vitreous samples from PDR patients (184.01±391.08 pg/ml), when compared to control (32.48±47.51 pg/ml; p = 0.005) or DM samples (25.39±36.92 pg/ml; p = 0.013). In comparison to the NPDR group no difference was detectable (29.19±50.36 pg/ml; p = 0.083; Fig 1C).

Findings in regard to Angiopoietin-2 were quite similar. An up-regulated Angiopoietin-2 expression was measured in PDR samples (1714.85±1194.35 pg/ml) from this study in contrast to the three other groups, namely controls (370.70±362.42 pg/ml; p<0.001), DM (638.47±828.36 pg/ml; p<0.001), and NPDR samples (492.46±719.90 pg/ml; p<0.001; Fig 1D).

### Cytokine up-regulation during DR progression

**Fig 2:**
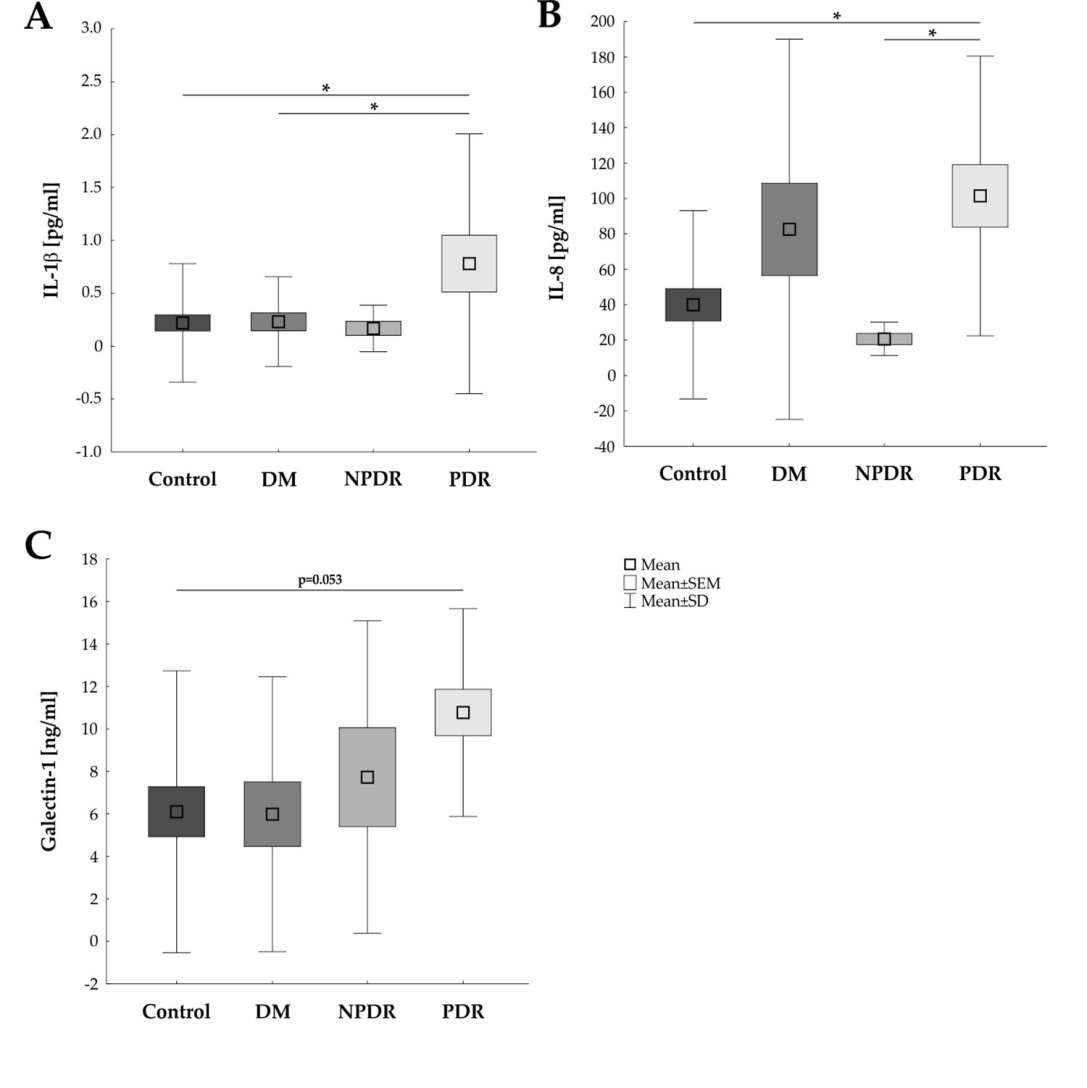
Increased pro-inflammatory cytokine expression in PDR. **A)** An increased IL-1β expression level was noted in PDR vitreous in contrast to control (p = 0.012) and DM (p = 0.043) samples. **B)** In addition, the IL-8 concentration was significantly higher in PDR patients than in control (p = 0.018) and NPDR patients (p = 0.034). **C)** Galectin-1 displayed a trend towards an up-regulation in PDR samples when compared to control samples (p = 0.053). DM: diabetes mellitus; NPDR: non-proliferative diabetic retinopathy; PDR: proliferative diabetic retinopathy; values are mean ± SEM ± SD; *p<0.05.

In addition to the examined angiogenic factors, an up-regulation of inflammatory cytokines could also be observed in the PDR group (Fig 2 and Table 3). The inflammatory cytokine IL-1β was elevated in PDR vitreous (0.78±1.23 pg/ml) when compared to control (0.22±0.56 pg/ml; p = 0.012) or DM samples (0.23±0.42 pg/ml; p = 0.043) but not the NPDR patients (0.17±0.22 pg/ml; p = 0.090; Fig 2A).

Additionally, the level of the inflamamtory cytokine IL-8 was up-regulated in the PDR patients (101.49±79.04 pg/ml) in comparison to the control (39.99±53.25 pg/ml; p = 0.018) and the NPDR (20.67±9.43 pg/ml; p = 0.034) but not the DM patients (82.59±107.41 pg/ml; p = 0858; Fig 2B).

Furthermore, a trend in up-regulation of Galectin-1, a member of the galactin family which is known for relatively broad specificity, could be observed in the PDR group (10.77±4.89 ng/ml) compared to the control group (6.10±6.64; p = 0.053). In comparison to the DM (5.99±6.47ng/ml; p = 0.099) and the NPDR group (7.73±7.36 ng/ml; p = 0.600) no differences were observable (Fig 2C).

In addition, the levels of the pro-inflammatory cytokines TNF-α and interferon gamma (INF-γ) were also analyzed but the expression level was under the detection level in all samples.

The inflammatory chemokine (C-C motif) ligand 3 (CCL3) was detctable in all vitreous samples but the expression level, which is regulated by TNF-α and IFN-γ, showed no differences between the control group, the DM, the NPDR, and the PDR group (Table 3).

### Correlation analysis

**Fig 3:**
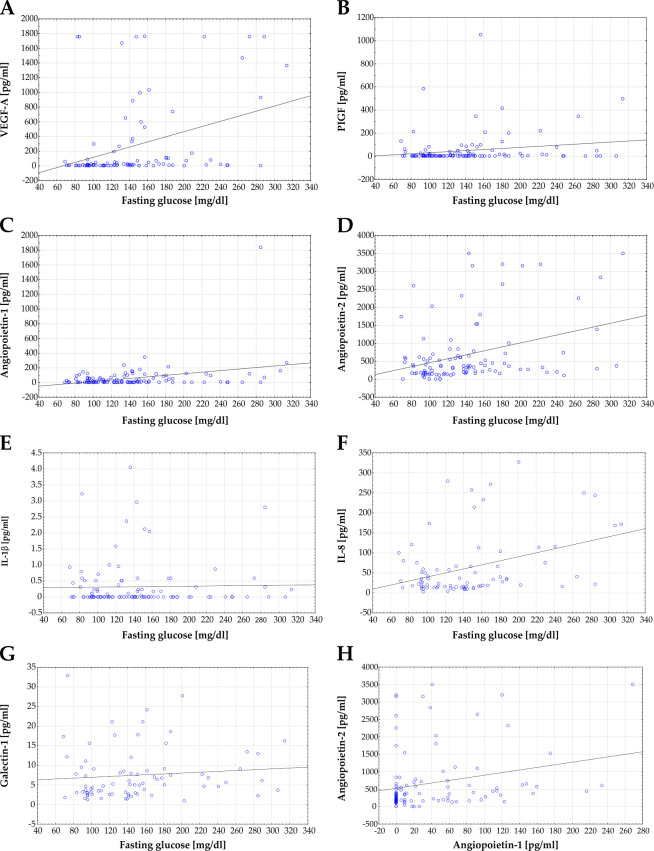
Correlation of protein concentration and fasting glucose level. **A)** Correlation analysis of VEGF-A concentration in vitreous humour and fasting glucose level revealed a significant correlation for all samples (r = 0.366; p<0.001; r^2^ = 0.134). **B)** Correlation analysis of PIGF concentration and fasting glucose also showed a significant correlation (r = 0.186; p = 0.046; r^2^ = 0.035). **C)** Scatterplot of Angiopoietin-1 versus fasting glucose. A significant correlation between Angiopoietin-1 level and fasting glucose level could be observed (r = 0.3275; p<0.001; r^2^ = 0.076). **D)** Also, a significant correlation was found between Angiopoietin-2 and fasting glucose level (r = 0.348; p<0.001; r^2^ = 0.121). **E)** A correlation between levels of IL-1β and fasting glucose could not be observed (r = 0.023; p = 0.813; r^2^ = 0.001). **F)** In contrast, a significant correlation between IL-8 and fasting glucose level was detectable (r = 0.374; p<0.001; r^2^ = 0,140). **G)** Scatterplot of Galectin-1 levels and fasting glucose levels revealed no correlation (r = 0.099; p = 0.386; r^2^ = 0.0098). **H)** A significant correlation between Angiopoietin-1 and Angiopoietin-2 levels were detectable, excluding one outlier patient (patient #52; r = 0.245; p = 0.011; r^2^ = 0.060). Each blue dot represents one patient, linear regressions are displayed as solid grey lines.

Correlation analysis of protein concentrations of several markers in vitreous samples and fasting glucose levels were performed. A significant correlation between levels of VEGF-A and fasting glucose was noted for all samples (r = 0.366; p<0.001; r^2^ = 0.134; Fig 3A). Additionally, a positive significant correlation was detectable for the PIGF concentration per patient and fasting glucose levels of these patients (r = 0.186; p = 0.046; r^2^ = 0.035; Fig 3B).

Analyzing the correlation between the angiopoietin members and the fasting glucose level revealed a significant correlation between Angiopoietin-1 and the fasting glucose value for all samples (r = 0.328; p<0.001; r^2^ = 0.076; Fig 3C). In addition, a significant correlation was seen for Angiopoietin-2 and fasting glucose (r = 0.348; p<0.001; r^2^ = 0.121; Fig 3D).

In contrast, no significant correlation was found between patients individual IL-1β level and fasting glucose levels (r = 0.023; p = 0.813; r^2^ = 0.001; Fig 3E). Whereas for IL-8, a significant correlation between IL-8 and fasting glucose levels was detectable (r = 0.374; p<0.001; r^2^ = 0.140; Fig 3F).

Furthermore, no significant correlation was found between the individual Galectin-1 concentrations and fasting glucose levels (r = 0.099; p = 0.386; r^2^ = 0.010, Fig 3G).

We additionally evaluated whether the level of Angiopoietin-1 level correlates with the level of Angiopoietin-2. We observed a significant correlation between both angiogenic factors (r = 0.245; p = 0.011; r^2^ = 0.060; Fig 3H).

In addition to the correlation analysis of the fasting glucose level and the protein concentrations in the vitreous, correlation analyses between VEGF-A as a key marker for DR and the other proteins were also carried out.

**Fig 4:**
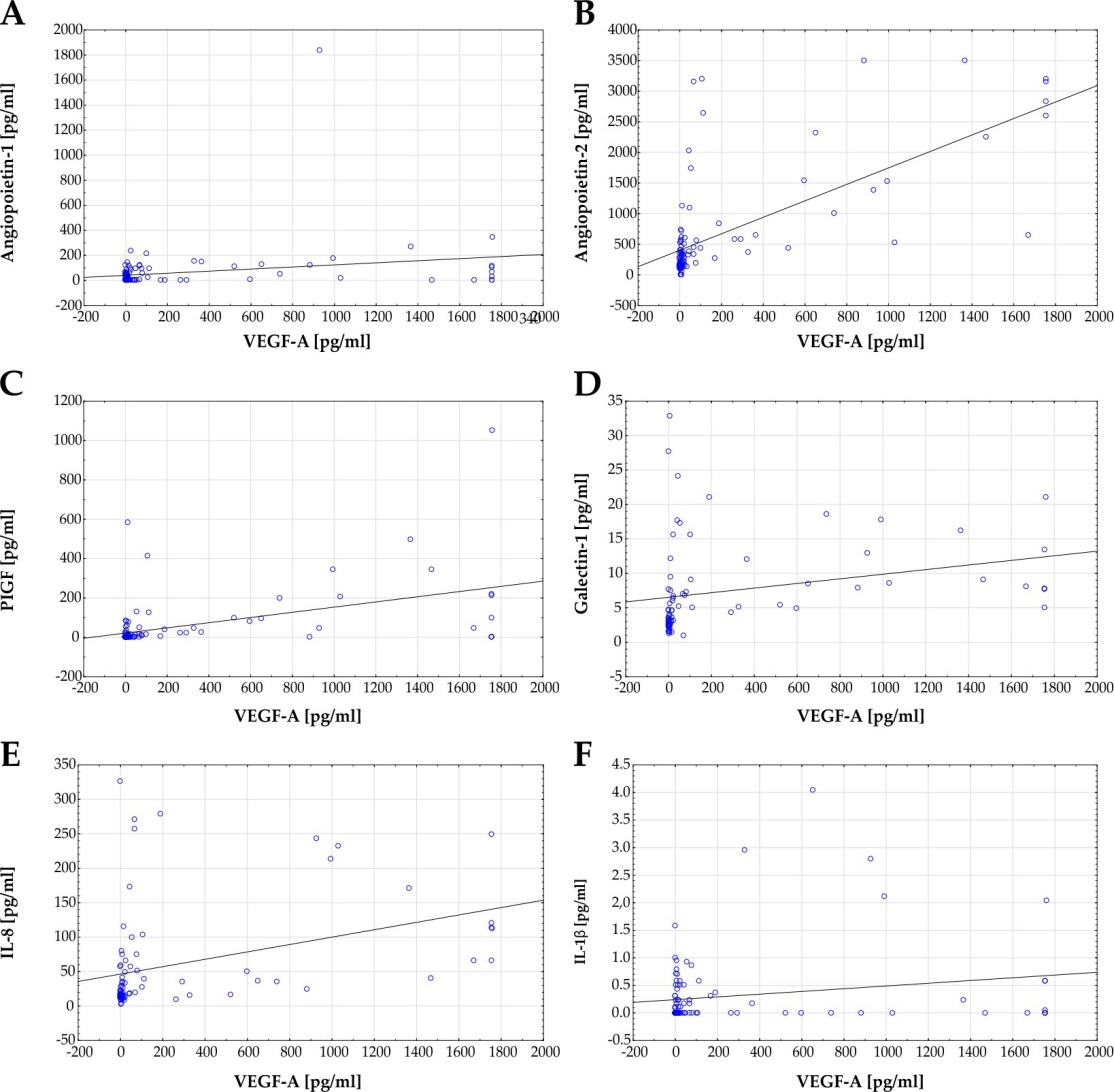
Correlation of VEGF-A levels with other protein concentrations. **A)** A significant correlation of Angiopoietin-1 and VEGF-A was noted for all samples (r = 0.228; p = 0.023; r^2^ = 0.052). **B)** Scatterplot of Angiopoietin-2 versus VEGF-A. A significant correlation between Angiopoietin-2 level and VEGF-A could be observed (r = 0.348; p<0.001; r^2^ = 0.121). **C)** Correlation analysis of VEGF-A and PIGF concentration also showed a significant correlation (r = 0.479; p < .001; r^2^ = 0.229). **D)** Also, between Galectin-1 and VEGF-A a significant correlation was detectable (r = 0.271; p = 0.019; r^2^ = 0.074). **E)** A significant correlation between IL-8 and VEGF-A was detectable (r = 0.371; p<0.001; r^2^ = 0.138). **F)** In contrast, a correlation between levels of IL-1β and VEGF-A could not be observed (r = 0.196; p = 0.051; r^2^ = 0.039). Each blue dot represents one patient, linear regressions are displayed as solid grey lines.

A significant correlation between levels of Angiopoietin-1 and VEGF-A (r = 0.228; p = 0.023; r^2^ = 0.052; Fig 4A) was observed. Moreover, a very strong correlation was noted between Angiopoietin-2 and VEGF-A (r = 0.348; p<0.001; r^2^ = 0.121; Fig 4B) as well as PIFG and VEGF-A (r = 0.479; p<0.001; r^2^ = 0.229; Fig 4C). In addition, level of Galectin-1 and VEGF-A (r = 0.271; p = 0.019; r^2^ = 0.074; Fig 4D) and IL-8 and VEGF-A showed a significant correlation (r = 0.371; p<0.001; r^2^ = 0.138; Fig 4E). In contrast no significant correlation was detectable between patients individual IL-1β and VEGF-A level (r = 0.196; p = 0.051; r^2^ = 0.039; Fig 4F).

## Discussion

DR is a sight-threatening condition that is set to increase worldwide over the next few decades due to the aging of society and the increase in DM. Anti-VEGF therapies are applied in case of PDR or diabetic macular edema, but frequent administration is required, and the macula edema of some patients does not respond to the various available anti VEGF therapies resulting in significant visual loss despite treatment [13, 33].

However, other candidates besides VEGF might contribute to disease progression. This led to the objective of this prospective study, which investigated which other factors are involved in the pathogenesis of DR and to what extent protein changes occur during DR progression. Therefore, in addition to VEGF, the level of other angiogenic factors and inflammatory cytokines in the aqueous humour between NDPR and PDR patients was directly compared in the present study.

Therefore, we evaluated protein alterations during different stages of DR of 116 patients undergoing pars plana vitrectomy. The control and DM patients were significantly older than the PDR patients. The acquisition of patients undergoing pars plana vitrectomy for indications other than DR may result in an age difference between the groups. Using patients who undergo a pars plana vitrectomy as a control group is an established approach [10, 34, 35], since otherwise only cadavers are available [7]. However, freshly obtained samples are preferable to post-mortem tissue/liquids, since the human tissue is getting worse with every post-mortem hour.

Our study data showed an up-regulation of VEGF-A in the group of PDR compared to other groups. In addition, the VEGF-A concentration was positively correlated with fasting glucose level. This result agrees with the past studies of VEGF concentrations in the fluids of the eye and the known role of VEGF in PDR [34]. The successful development of therapies to DR by targeting this factor supports this concept and encourage to search for other potentially active factors in the pathological process.

However, not only VEGF-A, but also the other examined angiogenic factors showed this up-regulation in the PDR group compared to the other groups. Thus, PIGF was significantly up-regulated in the PDR group and positively correlated with VEGF-A levels. This result is consistent with our previous study [12] as well as other studies examining this factor in the vitreous [36, 37]. PIGF assumes a major role in angiogenesis [38]. On the one hand, PIGF activates its own signaling via the VEGF receptor 1 (VEGFR-1) and on the other hand, it enhances the VEGFR-2 pathway in the direction of neovascularization by displacing VEGF-A from VEGFR-1 [39]. Therefore, the results of the study support the statement that PIGF, as an angiogenetic factor, seems to be involved in the progression of DR. Furthermore, our data support the assumption that several VEGF family members should be used as targets for PDR treatment in the future. In line, aflibercept, which binds both VEGF-A and PIGF, is already successfully used for the treatment of diabetic macula edema [40]. However, whether binding PIGF is actually the reason why aflibercept has an advantage in patients is not yet been conclusively proven.

In addition to the VEGF family, recent studies revealed that the angiopoietin family also plays a role in regulating blood vessel sprouting and growing. In this context, Angiopoietin-1 and -2 seems to act agonistic and antagonistic [15]. Angiopoietin-1 blocks diabetic damage dose-dependently to the blood-retina barrier and appears to be protective effect against DR [17]. In contrast, Angiopoietin-2 is expressed under pathological conditions, when VEGF levels are high and proinflammatory cytokines are expressed. Therefore, Angiopoietin-2 synergistically promotes vascular permeability and stimulates retinal neovascularization [18, 20], hence it is not surprising that Angiopoietin-2 was elevated in the serum of PDR patients [21, 41]. Interestingly, we found both Angiopoietin-1 and -2 up-regulated in the vitreous samples of the PDR patients compared to the other groups. In addition, a significant positive correlation between the Angiopoietin-1 and -2 levels, as well as a positive correlation between Angiopoietin-1 and VEGF-A as well as Angiopoietin-2 and VEGF-A could be detected. The data of Angiopoietin-2 are in line with a previous study which also demonstrated an increase of Angiopoietin-2 in the vitreous fluid of patients with PDR and suggested an association of Angiopoietin-2 and VEGF with angiogenic activity in PDR [42]. However, the strong up-regulation of Angiopoietin-1 levels in PDR patients could not confirm data from previous studies in animal models [43], the previously described up-regulation in the serum of NPDR patients [21] and its known antagonistic effect on Angiopoietin-2 [15]. The regulation of Angiopoietin-1 in the vitreous humor in DR has not yet been studied in detail. However, the data support the relevance of both members (Angiopoietin-1 and -2) as a target for DR therapy, although the function and role of Angiopoietin-1 as well its interaction with Angiopoietin-2 needs to be further investigated. The focus should be on clarifying whether the high level of Angiopoietin-1 represents an attempt to counteract the increased level of Angiopoietin-2 or itself represents a deleterious influence in PDR pathogenesis. The available data on Angiopoietin-2 inhibition to the therapeutic value still remains controversial.

The role of faricimab as a simultaneous inhibitor of Angiopoietin-2 and VEGF-A to treat diabetic macula edema is now established, and a commercial preparation is available [44]. The faricimab showed non inferiority in the treatment of diabetic macula edema compared to aflibercept and an anatomical and functional improvement with intervals up to 16 weeks [45].

On the other hand, nesvacumab, an Angiopoeitin-2 inhibitor, in combination with aflibercept revealed no vison improvement for patients with diabetic macula oedema in comparison to VEGF/PIGF inhibition monotherapy by aflibercept [46]. Therefore, the application of an Angiopoeitin-2 inhibitor for the treatment of DR needs to be further investigation and seems to be strongly dependent on the level of other factors such as VEGF but also Angiopoietin-1. It would thus be interesting to investigate whether a simultaneous modification of Angiopoietin-1 (inhibition/activation) and Angiopoietin-2 improves the success of the treatment.

Similar to the angiogenesis factors, we could find an upregulation of pro-inflammatory cytokines. It should be noted that IL-1β values were generally very low and close detection limit of the ELISA kits and in the PDR group near the detection limit. Nevertheless, this result is consistent with our previous study, in which we did not differentiate between NDPR and PDR patients [12]. In addition, other research groups were also able to find post mortem increased levels of IL-1β and its activator molecule caspase-1 in the vitreous body of patients with PDR [7].

Moreover, we could detect an up-regulation of IL-8 in comparison to the controls and the NPDR, but not the DM group, which in this case is most likely due to the strong scatter in the DM group. Moreover, for the first time we could find that the individual IL-8 concentrations significantly correlate with the fasting glucose levels. The up-regulation of IL-8 in vitreous PDR patients has already been mentioned [47], including a recent study by Loporchio et al. [35]. Interestingly, a prospective study by Yenihayat and colleagues revealed that a relatively small number of NPDR patients, showed a higher IL-8 concentration according to the presentation of subretinal fluids in the macula. However, in this study the VEGF levels showed no dependence on the sub-retinal fluid. This suggests that inflammation is an important factor in the progression of diabetic macular oedema, leading to subretinal fluid formation in diabetic patients [48].

Regarding the pro-inflammatory cytokines TNF-α and INF-γ, no expression was detectable with the used ELISA kits. There are contradictory statements in the literature regarding the importance of TNF-α as a vitreous marker for PDR. It was initially considered a marker [49, 50], but this could not be detected in the latest studies [35]. The same applies to INF-γ, which is postulated by some studies as a vitreous marker for PDR [50], and was not detectable in our case. Both markers seem to be subject to strong fluctuations and the importance of these cytokines in PDR should be determined in future studies.

Furthermore, the expression levels of the inflammatory chemokine CCL3 showed no differences between the four groups. This is consistent with the fact that TNF-α and INF-γ expression is not altered, since the expression of CCL3 is regulated by TNF-α and IFN-γ [51, 52].

Galectin-1 is responsible for the proper execution of the angiogenesis process also by activating the VEGFR-1 [23, 24, 53]. In our study, a positive correlation between Galectin-1 and VEGF-A levels as well as a trend toward an up-regulation in the PDR patients compared to the controls could be found. This finding is consistent with a study by Abu El-Asrar et al. who found a significant increase in Galectin-1 in vitreous samples of PDR patients compared to non-diabetics [22]. Furthermore, both Galectin-1 and VEGF are up-regulated in hypoxic microenvironments by HIF-dependent signalling [54], suggesting that Galectin-1 alongside VEGF seems to be an interesting marker to treat the disturbed vascularization in DR.

Thus, our study indicates pro-inflammatory cytokines as well as angiogenetic factors, which contribute to the pathogenesis of DR. Interestingly all analyzed proteins revealed alterations in PDR but not in NPDR patients. Hence, additional identifications of protein alterations in DR patients should be performed to identify NPDR specific alterations. For the PDR treatment in the future, both anti-inflammatory and anti-neovascularization agents could be used, possibly simultaneously. Therefore, the role of Angiopoietin-1 and Galectin-1 should be analyzed in more detail.

## Conclusion

Our study demonstrates the importance of angiogenic factors, besides VEGF, in the development of PDR. Angiopoietin-1 and -2 as well as the new marker Galectin-1 seem to represent an interesting starting point for further treatment development for DR. Furthermore, we were able to confirm an increase in the pro-inflammatory biomarker IL-8 in patients with PDR. Interestingly, all investigated pro-inflammatory factors were not elevated in the NPDR patients. The results highlight new potential avenues for targeted or additive therapies that can help to identify and treat severe complications of DR to save and improve vision.
